# Prenatal natural history of isolated fetal mild bilateral pyelectasis

**DOI:** 10.6061/clinics/2016(09)05

**Published:** 2016-09

**Authors:** Gustavo de Paula Pereira, Victor Bunduki, Eliane Azeka Hase, Rossana Pulcineli Vieira Francisco, Marcelo Zugaib

**Affiliations:** Faculdade de Medicina da Universidade de São Paulo, Departamento de Obstetrícia e Ginecologia, São Paulo/SP, Brazil

**Keywords:** Fetal Pyelectasis, Fetal Hydronephrosis, Renal Pelvis, Ultrasonography

## Abstract

**OBJECTIVE:**

To analyze the prenatal outcomes in a cohort of fetuses with mild bilateral pyelectasis and determine whether performing serial ultrasounds is a good follow-up strategy.

**METHODS:**

A prospective longitudinal study was conducted on 62 fetuses with mild bilateral pyelectasis. Fetal mild bilateral pyelectasis was considered when the renal pelvis measured (in millimeters) ≥5.0 to 10.0, ≥7.0 to 10.0, and ≥10.0 to 15 at ≤23 weeks 6 days, 24 to 31 weeks 6 days, and ≥32 weeks, respectively, with no uretero-calyceal dilatation. Ultrasounds were performed every 3 weeks to assess whether the mild bilateral pyelectasis regressed, remained unchanged (Group 1) or progressed (Group 2).

**RESULTS:**

Group 1 consisted of 53 fetuses (85.4%), and progression was observed in 9 cases (Group 2, 14.6%). The initial renal pelvis diameter was significantly larger in fetuses with progression (*p*=0.028). Statistically significant differences in the renal pelvis diameter were also found at weeks 31 and 35 for both kidneys (*p*<0.05). The cases requiring intrauterine procedures or early delivery were not observed.

**CONCLUSION:**

Fetal mild bilateral pyelectasis with no calyceal dilatation is a benign condition that can be managed in the postnatal period. The initial renal pelvis diameter and the diameter in week 31 or 35 were valuable parameters for identifying cases that would eventually need specific postnatal procedures.

## INTRODUCTION

Pyelectasys (PE), i.e., a dilatation exclusively involving the fetal renal pelvis, is a common ultrasonographic finding that is identified in 1-5% of fetuses in the second and third trimesters of pregnancy 1-8. Various criteria are used to define renal pelvis dilatation. The cut-off limits for defining mild pyelectasis also vary 9-12. Many of these cases are sent to referral centers and subjected to serial scans; however, these interventions do not have a significant impact on prognosis, in accordance with the literature, which characterizes PE as regressive with self-limiting lesions 11-18. However, in some cases, lesions can progress and become an issue in perinatal care.

Identifying PE cases that can progress to medium or severe dilatation with hydronephrosis, characterized by an altered amount of amniotic fluid and possible renal function impairment, is important for determining appropriate perinatal care strategies. Thus, knowing the natural prenatal history and identifying eventual progression factors of FMBP cases are of great interest because serial ultrasound scanning can lead to unnecessary expenses and anxiety. A better understanding of FMBP could also eliminate the dissemination of equivocal information to parents and provide them with better support 5,. However, in cases with potential renal disease, it is possible to perform a suitable diagnostic or therapeutic intervention and prevent kidney function damage 22-24.

Therefore, the aims of this study were to analyze the prenatal outcomes in a cohort of fetuses with mild bilateral pyelectasis (FMBP) and determine whether performing serial ultrasounds (USs) is a good follow-up strategy.

## MATERIALS AND METHODS

A prospective study of a cohort of fetuses with FMBP referred to our center was carried out between June 2011 and December 2012 at the Fetal Medicine Unit at São Paulo University Medical School Hospital, Brazil.

FMBP was defined by an RP diameter of ≥5.0 mm and <10.0 mm until 23 w 6 d; ≥7.0 mm and <10.0 mm between 24 w and 31 w 6 d; and ≥10.0 mm and <15.0 mm from 32 w gestational age (GA) with no uretero-calyceal dilatation 25-29.

The inclusion criteria were singleton pregnancies with FMBP according to the above criteria, absence of calyceal dilatation, and absence of ultrasonographic signs of lower urinary tract obstruction, i.e., absence of urethral and/or bladder dilatation at the time of pyelectasis diagnosis.

The exclusion criteria were less than three USs performed at our center, diabetic patients, macrosomic fetuses (fetal weight over the 90^th^ percentile) or intrauterine growth-restricted fetuses (estimated fetal weight under the 10^th^ percentile) 30, fetal chromosomal defects, fetal death during follow-up, and the presence of other fetal structural abnormalities.

Cardiac defects and other structural malformations were ruled out with a fetal structural ultrasound and echocardiography.

Ultrasound examinations were performed every three weeks. The serial assessments of the fetal renal pelvis were performed by measuring the anteroposterior diameter of each renal pelvis in millimeters to one decimal in a strict transverse view of the fetal abdomen at the level of the renal pelvis, preferably with the spine in the anterior position, and by the visualization of symmetric lateral ossification centers. The presence or absence of visible calyceal groups was confirmed by coronal US views of the kidneys at the level of the renal pelvis.

### Natural history

For the entire cohort of fetuses, multiple Bonferroni comparisons 31 between the diameters until the 24th week and the RP diameters obtained after the 24th week (on a week-by-week basis) were performed to determine the natural evolution of RP diameters over subsequent weeks compared with the diameters in earlier weeks (gestational age when fetal structural US is routinely performed). The left and right kidneys were analyzed separately.

The first and last assessments of RP diameters were then compared with the RP diameters of the entire population using paired Student's t tests 32 to assess the natural history of FMBP.

### Progression, stability, and regression analysis

FMBP progression, regression, and stability were defined as follows.

- Regression: measurements returning to the normal range for the corresponding gestational age.
- Progression: fetal renal pelvic diameter becoming greater than the reference values for at least one kidney ?? or the presence of calyceal dilatation.
- Stability: RP diameter being maintained at the same level according to gestational age for at least one kidney.

Based on the last prenatal US examination, the cases were divided into two groups: regression and stability (Group 1) and progression (Group 2). The results were compared between groups.

Maternal characteristics (age and parity), initial RP diameter, GA at diagnosis, and fetal gender were also assessed and compared between groups (mean, standard deviation, median, minimum, and maximum) using Student's t test or the Mann-Whitney U test and chi-square test (fetal gender) to determine whether any of these variables could distinguish between the different groups.

## ETHICS

This study followed the tenets of the Declaration of Helsinki and the rules of Resolution No. 196/96 of the Brazilian National Health Council. All patients were informed about the research objectives. Only those who voluntarily agreed to participate by signing a Statement of Informed Consent were included.

## RESULTS

### Sample Characteristics

A total of 62 fetuses met the inclusion criteria and had a complete set of follow-up USs during the prenatal period. Initially, 73 cases were included in the analysis. However, 11 patients were excluded for the following reasons: missed follow-up in 7 cases, chromosomal abnormalities in 2 cases (T21), and gestational diabetes in 2 cases.

The fetuses population consisted of 47 males and 15 females (3.1:1). The gestational age at diagnosis ranged from 19.2 to 30.1 weeks (mean 23.2 weeks). The total number of US examinations was between 3 and 7 (mean 4.5).

The maternal age ranged from 15 to 44 years (mean 28.1 years). The parity ranged between 1 and 10 pregnancies (mean 2.1 pregnancies).

### Natural History

**Figure 1:**
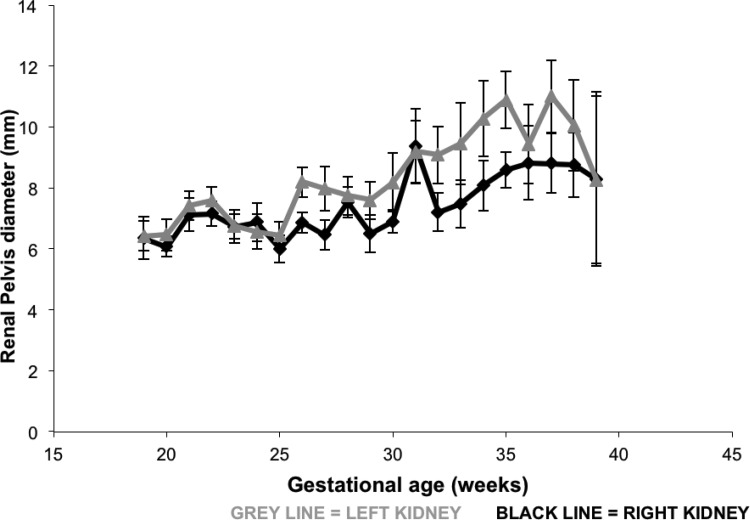
Mean profiles and standard errors of the RP diameters according to gestational age.

There was a significant difference in the mean RP diameter between the gestational weeks in both kidneys (*p*<0.001) (Figure 1).

Although the RP diameters increased in the remaining weeks of pregnancy compared with the first weeks (19 to 24 weeks), the only statistically significant differences were in gestational weeks 31 and 35 for both kidneys (*p*<0.05). However, these differences were not maintained every week or after a certain gestational age (*p*>0.05) (Table 1).

For both kidneys, there was a statistically significant average increase in the RP diameters from the first to the last assessment (*p*=0.001 and *p*<0.001, respectively), with the first evaluation performed between 19 and 30 weeks of pregnancy and the last assessment performed between 30 and 40 weeks gestation (Table 2).

The volume of amniotic fluid remained normal in the ultrasound evaluations in all the cases.

### Groups

During the prenatal period, pyelectasis regression occurred in 29 cases (46.7%), i.e., there was a normalization of the RP without calyceal dilatation. In 24 cases (38.7%), dilatation of the RP remained stable (pyelectais remained in at least one kidney). Stability and regression (Group 1) occurred in 53 cases (85.4%).

Unilateral progression (Group 2) was observed in 9 cases (14.6%). Severe cases, i.e., cases that required intrauterine procedures or early delivery, were not observed in this cohort.

The only statistically significant difference between the groups was the initial RP diameter of the right kidney, which was, on average, larger in the fetuses in Group 2 (*p*=0.028). Although not statistically significant, there was a bigger initial diameter of the left kidney pelvis in the Group 2 fetuses (*p*=0.116) (Table 3).

Maternal age, parity, gestational age at diagnosis, and fetal gender were not significantly different between the groups.

### PERINATAL RESULTS

Thirty-two newborns (NBs) in this prenatal cohort were examined. Only the first postnatal US was considered in the analysis. The NB age at the time of ultrasound varied from 4 to 20 days (mean 7.3 days).

Twenty-six NBs were in the regression or stability prenatal group. Twenty had normal kidneys at the postnatal ultrasound. Six showed persistent mild pyelectasis, with no clinical consequences to date. Six NBs were in Group 2, three of whom had no clinical events and three of whom were followed in the nephro-urologic unit. The NB analysis results were not discordant with the Group 1 or 2 prenatally classified fetuses.

## DISCUSSION

The goal of this study was to assess the natural history of FMBP and to investigate whether serial US scans are needed in cases of isolated pyelectasis without calyceal dilatation.

This series consisted of a prospective study in a cohort of fetuses with MBPE with rigorous inclusion criteria; i.e., only bilateral pyelectasis cases with no calyceal dilatation were included.

The study results showed that the RP diameters in the cases of FMBP increased in late pregnancy compared with the second trimester. The differences were statistically significant for both kidneys in gestational weeks 31 and 35. An increase in the RP diameters from the first to the last assessment was also demonstrated. This observation is similar to results in the literature. The relationship of the RP diameter with postnatal prognosis has also been confirmed 5,13,21,27,33-35.

After the initial analysis, the cases were divided into two groups: the stable or regression group (85.4%) and the progression group (14.6%). The literature has shown that the outcomes are essentially benign. More than 85% of RP dilatation cases remain stable or regressed 9,20,26,27,33,36,37. In the present series, even in cases in which progression was observed, there were no severe cases, i.e., no cases requiring early delivery or invasive intrauterine procedures. Additionally, no changes in amniotic fluid levels were observed. Repeating US scans every three weeks did not show any benefit in our study because no intervention was indicated in the prenatal period.

Although the number of cases in Group 2 was not large, some findings were of interest when we compared the groups. For example, the initial pelvic diameter was significantly higher in Group 2 (for the right kidney). This result suggests that the higher the RP diameter at the time of diagnosis, the greater the possibility that the renal pelvis will remain dilated during pregnancy, which is consistent with the literature 21,34,38.

Based on the natural history obtained in the present study, the diameters of the renal pelvis increased in late pregnancy compared with the weeks in which the structural fetal ultrasound was performed (19 to 24 weeks), reaching a statistically significant difference in gestational weeks 31 and 35. Based on these findings, we suggest that such cases can be followed in routine primary prenatal care. Only a final third trimester scan could be offered at a referral center to inform postnatal care strategies. Another point to consider is that the characterization of the natural history of FMBP enables appropriate counseling of parents facing this fetal diagnosis.

The selection criteria in this series were defined to ensure that cases with true urinary tract abnormalities were excluded. Thus, no cases with a RP diameter over 10 mm at 23 w 6 d and 31 w 6 d, as well as no cases with a RP diameter over 15 mm after 31 w 6 d were included. It is known that these grades of dilatation correlate well with a worse prognosis in the postnatal period 13,17,21,38,39.

The criteria used to classify antenatal hydronephrosis are often different from those used in the present study and are not uniform in the literature 9-11,40. Many authors define selection criteria based only on the pelvic diameter without taking into account the calyceal pattern 5,27,41. For these reasons, a comparison of the present series with other literature series is difficult.

To our knowledge the strategy of performing a serial US in the FMBP cases has not previously been prospectively assessed in the literature. To assess the cost of performing serial prenatal USs, Yamamura et al. 13 have concluded, based on a retrospective series, that a third trimester scan can be offered after the second trimester diagnosis of FMBP. This strategy would reduce costs and would not change the prenatal or perinatal outcomes.

Thus, the data from this study are clinically relevant because they demonstrate that no prenatal procedures are needed for FMBP and referral center care, and serial US scans can be avoided. Our results show that unnecessary overbooking of referral centers can be avoided to keep prenatal and childbirth care at low-risk centers in many instances. In addition, the proposed prenatal follow-up would lower the costs of prenatal care because only routine US examinations would be required.

Although the goal of this study was to determine the prenatal evolution of FMBP, some postnatal results were obtained. Thirty-two NBs in the cohort were examined based only on the first postnatal US in the follow-up period. The NB age at the time of US varied from 4 to 20 days (mean 7.3 days). Twenty-six NBs were in Group 1. Twenty had normal kidneys, and six showed persistent mild pyelectasis with no clinical consequences to date. Six NBs were in Group 2, three of whom had no clinical events and three of whom were followed in the nephro-urologic unit. In this small neonate group, no discordance was found between the prenatal classification and postnatal findings.

As a final comment, in this study, sample size estimation was not possible despite the prospective nature of the study because there are not enough data in the literature. In future studies, an accurate estimation of sample size could be obtained based on a 15% estimated rate of cases presenting with progressive FMBP. The proposed strategy of performing only one US scan late in the third trimester could be compared with serial prenatal US in cases of FMBP in a paired, randomized prospective trial.

## AUTHOR CONTRIBUTIONS

Pereira GP is the principal investigator, provided substantial contributions to the conception and design of the study, was responsible for the data acquisition, analysis and interpretation, manuscript draft, critical revision of the manuscript for important intellectual content and final approval of the version to be published. Bunduki V and Hase EA provided substantial contributions to the conception or design of the study and were responsible for the data interpretation, critical revision of the manuscript for important intellectual content and final approval of the version to be published. Francisco RP and Zugaib M were responsible for the approval of the manuscript final version.

## Figures and Tables

**Table 1:** Results of multiple Bonferroni comparisons of the renal pelvic diameters before 25 weeks gestation with those in the other weeks.

Kidney	Comparison		Mean Difference	Standard Error	df	*p*	CI (95%)	
							Lower	Upper
Right	19 to 24 weeks -	25 weeks	0.31	0.58	1	>0.999	-1.73	2.35
	19 to 24 weeks -	26 weeks	-0.18	0.47	1	>0.999	-1.85	1.50
	19 to 24 weeks -	27 weeks	-0.18	0.51	1	>0.999	-1.96	1.60
	19 to 24 weeks -	28 weeks	-1.06	0.57	1	>0.999	-3.07	0.95
	19 to 24 weeks -	29 weeks	-0.02	0.53	1	>0.999	-1.89	1.86
	19 to 24 weeks -	30 weeks	-0.20	0.52	1	>0.999	-2.05	1.65
	19 to 24 weeks -	31 weeks	-2.72	0.59	1	**<0.001**	-4.80	-0.65
	19 to 24 weeks -	32 weeks	-0.50	0.57	1	>0.999	-2.53	1.53
	19 to 24 weeks -	33 weeks	-1.10	0.56	1	>0.999	-3.07	0.87
	19 to 24 weeks -	34 weeks	-0.46	0.62	1	>0.999	-2.65	1.74
	19 to 24 weeks -	35 weeks	-2.29	0.58	1	**0.009**	-4.34	-0.24
	19 to 24 weeks -	36 weeks	-2.35	0.67	1	0.059	-4.72	0.03
	19 to 24 weeks -	37 weeks	-1.80	0.60	1	0.331	-3.93	0.32
	19 to 24 weeks -	38 weeks	-2.78	0.89	1	0.222	-5.94	0.37
	19 to 24 weeks -	39 weeks	-1.18	1.27	1	>0.999	-5.66	3.30
Left	19 to 24 weeks -	25 weeks	-0.68	0.69	1	>0.999	-3.12	1.75
	19 to 24 weeks -	26 weeks	-1.15	0.56	1	>0.999	-3.14	0.85
	19 to 24 weeks -	27 weeks	-1.41	0.60	1	>0.999	-3.53	0.72
	19 to 24 weeks -	28 weeks	-1.55	0.68	1	>0.999	-3.95	0.85
	19 to 24 weeks -	29 weeks	-1.08	0.64	1	>0.999	-3.33	1.17
	19 to 24 weeks -	30 weeks	-1.49	0.63	1	>0.999	-3.72	0.73
	19 to 24 weeks -	31 weeks	-2.57	0.71	1	**0.032**	-5.07	-0.08
	19 to 24 weeks -	32 weeks	-1.79	0.69	1	>0.999	-4.23	0.64
	19 to 24 weeks -	33 weeks	-2.90	0.67	1	**0.002**	-5.28	-0.52
	19 to 24 weeks -	34 weeks	-2.64	0.75	1	0.056	-5.30	0.02
	19 to 24 weeks -	35 weeks	-3.60	0.71	1	**<0.001**	-6.09	-1.11
	19 to 24 weeks -	36 weeks	-2.73	0.82	1	0.113	-5.64	0.18
	19 to 24 weeks -	37 weeks	-3.34	0.74	1	**0.001**	-5.96	-0.71
	19 to 24 weeks -	38 weeks	-3.64	1.10	1	0.109	-7.51	0.23
	19 to 24 weeks -	39 weeks	-2.02	1.55	1	>0.999	-7.48	3.44

Results of multiple Bonferroni comparisons.

**Table 2:** The first and last diameters of the renal pelvis and results of the comparative tests.

Variable	Evaluation	Average	SD	Median	Minimum	Maximum	N	*p*
Diameter of the pelvis of the right kidney	First	6.87	1.39	7	5	10	62	0.001
	Last	8.40	3.76	7.9	3	23.6	62	
Diameter of the pelvis of the left kidney	First	7.16	1.56	7.1	5	10	62	<0.001
	Last	9.59	4.84	9.2	2.2	23	62	
Gestational age (completed weeks)	First	22.74	2.70	22	19	30	62	
	Last	35.95	2.17	36.5	30	40	62	

Results of paired Student’s t tests.

**Table 3:** Description of the gestational age at diagnosis, initial diameters of the renal pelvis, maternal characteristics (age and pregnancies), and fetal gender according to the prenatal outcome* and results of statistical tests.

Variable	Prenatal outcome	Mean	SD	Median	Minimum	Maximum	N	*p*
GA (weeks)	Stability/Regression	23.17	2.54	22.86	19.29	29.14	53	0.901
	Progression	23.05	3.31	22.00	20.43	30.14	9	
	Total	23.15	2.64	22.64	19.29	30.14	62	
Diameter of the pelvis of the right kidney (mm)	Stability/Regression	6.72	1.33	7	5	9.8	53	**0.028**
	Progression	7.81	1.48	7.8	5	10	9	
	Total	6.87	1.39	7	5	10	62	
Diameter of the pelvis of the left kidney (mm)	Stability/Regression	7.03	1.51	7	5	10	53	0.116
	Progression	7.91	1.72	8	5	10	9	
	Total	7.16	1.56	7.1	5	10	62	
Maternal age (years)	Stability/Regression	28.11	7.29	28	15	44	53	0.999
	Progression	28.11	8.05	25	17	44	9	
	Total	28.11	7.34	27.5	15	44	62	
Pregnancies	Stability/Regression	2.15	1.43	2	1	7	53	0.438*
	Progression	2.44	2.96	1	1	10	9	
	Total	2.19	1.71	2	1	10	62	
Fetal gender (male)N (%)	Stability/Regression	40 (75.5)					53	>0.999**
	Progression	7 (77.8)					9	
	Total	47 (75.8)					62	

Results of Student’s t-tests

* Results of Mann-Whitney tests

** Results of chi-square tests.
